# Dietary Adjustments to Altitude Training in Elite Endurance Athletes; Impact of a Randomized Clinical Trial With Antioxidant-Rich Foods

**DOI:** 10.3389/fspor.2020.00106

**Published:** 2020-08-26

**Authors:** Anu E. Koivisto-Mørk, Ingvild Paur, Gøran Paulsen, Ina Garthe, Truls Raastad, Nasser E. Bastani, Rune Blomhoff, Siv K. Bøhn

**Affiliations:** ^1^Norwegian Olympic Sports Centre, Norwegian Olympic and Paralympic Committee and Confederation of Sports, Oslo, Norway; ^2^Norwegian National Advisory Unit on Disease-Related Undernutrition, Oslo University Hospital, Oslo, Norway; ^3^Department of Physical Performance, Norwegian School of Sport Sciences, Oslo, Norway; ^4^Department of Nutrition, Institute of Basic Medical Sciences, University of Oslo, Oslo, Norway; ^5^Division of Cancer Medicine, Oslo University Hospital, Oslo, Norway; ^6^Faculty of Chemistry, Biotechnology and Food Sciences, Norwegian University of Life Sciences, Ås, Norway

**Keywords:** nutrition, hypoxia, dietary intervention, carbohydrate, dietary assessment, altitude training, oxidative stress

## Abstract

**Background:** Altitude training stresses several physiological and metabolic processes and alters the dietary needs of the athletes. International Olympic Committee (IOC)'s Nutrition Expert Group suggests that athletes should increase intake of energy, carbohydrate, iron, fluid, and antioxidant-rich foods while training at altitude.

**Objective:** We investigated whether athletes adjust their dietary intake according to the IOC's altitude-specific dietary recommendations, and whether an in-between meal intervention with antioxidant-rich foods altered the athletes' dietary composition and nutrition-related blood parameters (mineral, vitamin, carotenoid, and hormone concentrations).

**Design:** The dietary adjustments to altitude training (3 weeks at 2,320 m) were determined for 31 elite endurance athletes (23 ± 5 years, 23 males, 8 females) by six interviewer-administered 24-h dietary recalls on non-consecutive days; three before and during the altitude camp. The additional effect of in -between meal intervention with eucaloric antioxidant-rich or control snacks (1,000 kcal/day) was tested in a randomized controlled trial with parallel design.

**Results:** At altitude the athletes increased their energy intake by 35% (1,430 ± 630 kcal/day, *p* < 0.001), the provided snacks accounting for 70% of this increase. Carbohydrate intake increased from 6.5 ± 1.8 g/kg body weight (BW) (50 E%) to 9.3 ± 2.1 g/kg BW (53 E%) (*p* < 0.001), with no difference between the antioxidant and control group. Dietary iron, fluid, and antioxidant-rich food intake increased by 37, 38, and 104%, respectively, in the whole cohort. The intervention group had larger increases in polyunsaturated fatty acids (PUFA), ω3 PUFA (n-3 fatty acids), ω6 PUFA (n-6 fatty acids), fiber, vitamin C, folic acid, and copper intake, while protein intake increased more among the controls, reflecting the nutritional content of the snacks. Changes in the measured blood minerals, vitamins, and hormones were not differentially affected by the intervention except for the carotenoid; zeaxanthin, which increased more in the intervention group (*p* < 0.001).

**Conclusions:** Experienced elite endurance athletes increased their daily energy, carbohydrate, iron, fluid, and antioxidant-rich food intake during a 3-week training camp at moderate altitude meeting most of the altitude-specific dietary recommendations. The intervention with antioxidant-rich snacks improved the composition of the athletes' diets but had minimal impact on the measured nutrition-related blood parameters.

**Clinical Trial Registry Number:** NCT03088891 (www.clinicaltrials.gov), Norwegian registry number: 626539 (https://rekportalen.no/).

## Introduction

Athletes who utilize terrestrial altitude training usually reside at 1,800–2,500 m above sea level (Bartsch and Saltin, 2008). The difference in partial pressure of oxygen at this altitude stimulates both acute and gradual physiological adaptive processes, like acute altitude-induced diuresis (Siebenmann et al., 2017) and increased ventilatory rate, as well as increased erythropoietin production leading to increased production of red blood cells and larger hemoglobin mass (hbmass) (Mazzeo, 2008). Hypoxia of a given magnitude also increases resting metabolic rate (RMR) (Butterfield et al., 1992; Woods et al., 2017b), although not all studies in athletes have come to the same conclusion (Woods et al., 2017a). Training in hypoxia elevates oxidative stress in blood (Dosek et al., 2007; Pialoux et al., 2009) and potentially also alters substrate utilization during exercise (Griffiths et al., 2019; Young et al., 2019). Accordingly, this environmental stress will also modify the dietary needs of the athletes which seem to require certain dietary adjustments for optimal hypoxic adaptations (Stellingwerff et al., 2019).

Current dietary recommendations for athletes at altitude cover several nutritional aspects (Maughan and Burke, 2012; Stellingwerff et al., 2014, 2019; Michalczyk et al., 2016). Emerging evidence shows that adequate energy availability prior to training at altitude is a perquisite for optimal hbmass increase (Heikura et al., 2018). Similarly, adequate energy intake that matches both the augmented energy requirements of training hours as well as the potentially increased RMR to maintain adequate energy availability and stable body weight is essential. Suppressed appetite at altitude may complicate the execution of this task (Butterfield, 1999). Dietary protein requirements at altitude are secondary to energy requirements because energy deficit alone leads to negative protein balance and loss of muscle mass at altitude (Butterfield, 1999). Although there is some evidence for hypoxia-induced reduction in protein synthesis (Rennie et al., 1983), this is observed at altitudes much higher (>5,000 m) than those used by modern athletes (Narici and Kayser, 1995), thus current protein recommendations for athletes training at moderate altitude do not deviate from those at sea level. The enhanced erythropoiesis at altitude abruptly elevates the need for iron. Iron stores prior to altitude sojourns need to be adequate (Govus et al., 2015), although it is still unclear what the ideal ferritin cut-off value is. Nevertheless, some iron supplementation seems necessary for optimal erythropoiesis regardless of the pre-altitude ferritin (Garvican-Lewis et al., 2016a). Larger carbohydrate consumption may be required to match the fuel requirements of increased training loads and the potentially higher carbohydrate oxidation rate at altitude (Brooks et al., 1991; Stellingwerff et al., 2014). Because of dry air and increased ventilation at altitude, adequate fluid intake is a prerequisite to compensate for the higher water losses (Michalczyk et al., 2016).

Reactive oxygen species (ROS) and nitrogen compounds that are continuously formed as a result of normal aerobic cellular metabolism increase with increasing energy expenditure. These reactive compounds are important for the cellular adaptive responses to training and induce endogenous antioxidant defense systems. However, too high levels of ROS that are not counterbalanced may lead to oxidative stress and damage lipids, proteins and DNA (Sies, 1997; Pingitore et al., 2015). Sudden increase in training load and/or training intensity together with altitude exposure increases oxidative stress and circulating inflammatory biomarkers (Lewis et al., 2015; Debevec et al., 2017; Koivisto et al., 2019). Accordingly, larger intake of antioxidants has been recommended (Michalczyk et al., 2016; Koivisto et al., 2019; Stellingwerff et al., 2019). However, because high-dose antioxidant supplementation has shown to have negative effects on training adaptation (Merry and Ristow, 2016), possibly due to interruption of normal ROS signaling (Merry and Ristow, 2016), plant-based foods is the recommended antioxidant source (Maughan and Burke, 2012). Also, as plant foods contain thousands of potential biologically active substances the beneficial health effects of plant-based foods may go beyond a direct antioxidant effect involving modulation of stress- and defense-related gene expression that are important for maintenance of cellular functions (Bøhn et al., 2010).

A recent comprehensive narrative review highlighted six major nutrition themes for athletes training at moderate altitudes: energy, iron, carbohydrate, hydration, and antioxidant requirements along with various ergogenic aids (Stellingwerff et al., 2019). It was concluded that there is a lack of research on altitude related nutrition at moderate altitudes (~1,600–2,400 m). Although several studies have investigated the effects of particular nutrients e.g., iron (Garvican-Lewis et al., 2018; Hall et al., 2019), to our knowledge, no study has described in detail whether endurance athletes adjust their dietary intake following ascend to moderate altitude. Furthermore, no previous study has investigated whether manipulating in-between meals can affect the nutritional status of elite athletes training at moderate altitude.

Thus, the aim of the present study was to investigate whether elite endurance athletes adjust their dietary intake according to IOC's dietary recommendations for altitude training (Maughan and Burke, 2012) during a 3-week altitude training camp (2,320 m), and whether a food-based antioxidant intervention, served as in-between meal snacks, has an impact on the athletes' dietary macro-and micronutrient composition and nutrition-related blood parameters.

## Subjects and Methods

This study, ClinicalTrials.gov (NCT03088891), was approved by the Norwegian Regional Ethics Committee (REK number 626539) 12th October 2015. All participants provided written informed consent after receiving comprehensive oral and written information about the project protocol following the formal enrollment.

### Study Design

**Figure 1:**
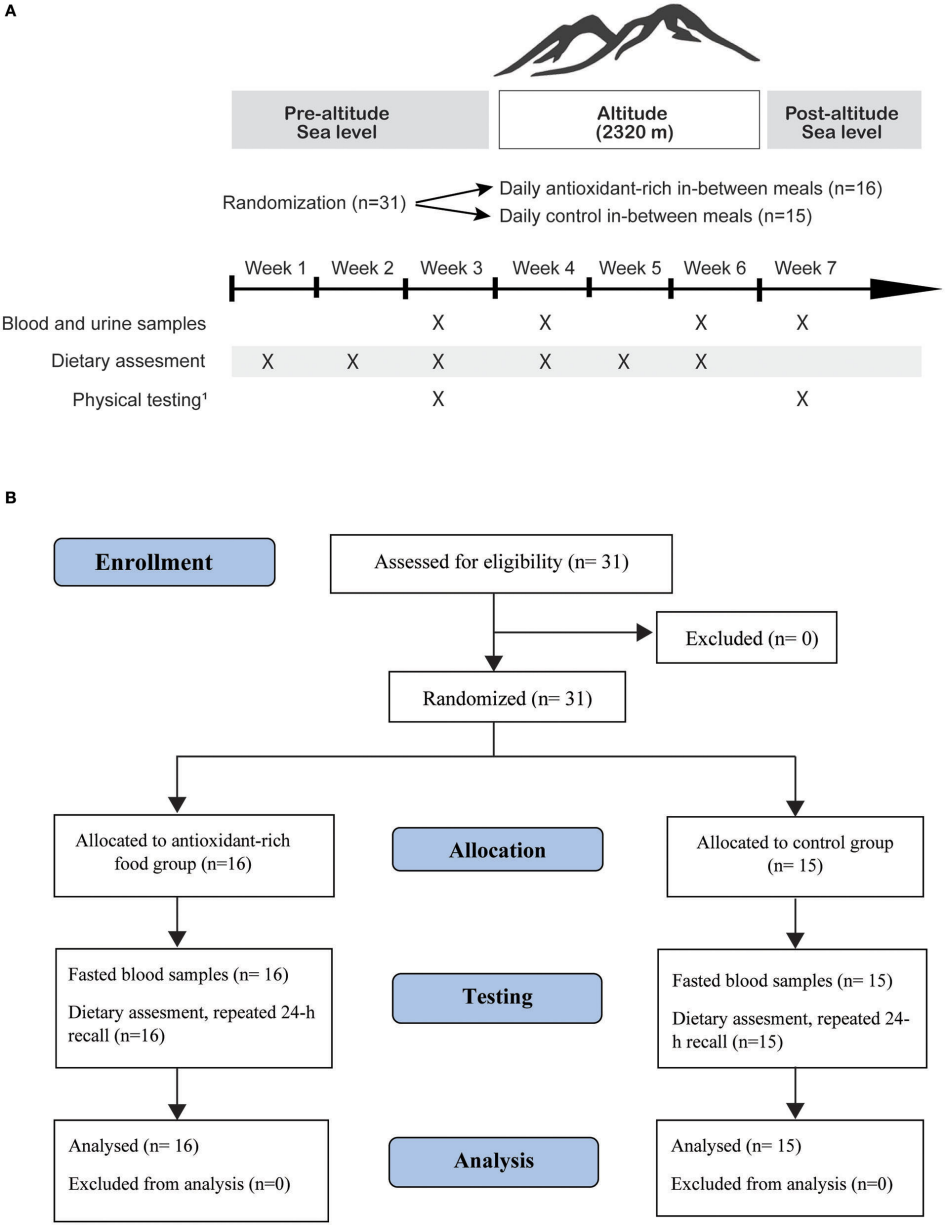
Experimental design and timeline of testing before, during and after the 3-week altitude training camp at 2,320 m above the sea level **(A)**, and Consort flow chart of the study participants **(B)**. ^1^Includes DXA, VO_2max_, hbmass, 100 m swimming as previously described (Koivisto et al., 2018).

The current study is part of a parallel randomized clinical trial (RCT) designed to assess the impact of a dietary intervention with antioxidant-rich foods on adaptations to altitude training in Norwegian elite athletes (Koivisto et al., 2018, 2019). The RCT, with allocation ratio 1:1, was conducted in October–November 2015, during the athletes' general preparation phase, less than a year prior to the Rio 2016 Olympic and Paralympic Games. Detailed description of the RCT is published previously (Koivisto et al., 2018). Data before and after the altitude camp were collected at the Norwegian Olympic Sports Center, Oslo, Norway while data collection during the 3-week altitude camp took place at the “High Altitude Training Centre” [Centro di Alto Rendimiento (CAR)] in Sierra Nevada, Spain (2,320 m). During the altitude training camp, athletes followed their respective National teams' training programs, logged their training, lived, and consumed their main meals at CAR (see Figure 1 for the timeline and experimental design of the study and Consort flow chart of the study participants). The average hypoxic dose for the study participants was 1,026 km h (Garvican-Lewis et al., 2016b).

In total, 31 national team endurance athletes from different sports attending the yearly altitude training camp were recruited to this study (females *n* = 8, males = 23; Paralympic athletes = 4, Olympic athletes = 27), including seven World Championship medalists. All invited athletes agreed to participate. Participants were randomly allocated to receive either antioxidant-rich foods (*n* = 16) or eucaloric control foods (*n* = 15) with significantly lower antioxidant content as in-between meals. The rationale for choosing a control-diet in the current design, instead of letting the control group consume snacks *ad libitum*, was to control for the effect associated with receiving a daily food-bag (the intervention snacks) from the investigator, as well as controlling for the extra energy provided. Each participant was given a random identification number prior to randomization. Randomization to intervention or control croup was performed using computer generated random sequence stratified by sport and gender. The researcher who performed the randomization was not involved in the participant enrolment or group allocation. All researchers involved in testing and sample analysis were blinded to group allocation except the researcher who performed the 24 h recalls and organized the practical delivery of food items to the participants.

### Food Environment and Dietary Intake

The study participants could freely choose their preferred meals throughout the day, apart from the daily intervention and control in-between meal snacks. Breakfast, lunch and dinner were provided by the cafeteria at CAR following their regular 3-week menu. The cafeteria menu was not influenced in any way during the study. The menu was varied and consisted usually of a salad buffet, several options for starters (e.g., vegetable soup, pasta), main courses (e.g., grilled chicken breast, baked halibut) as well as a side dish (e.g., steamed spinach, baked eggplant) and dessert (e.g., pudding, cake, fresh fruit, yogurt). All participants could also purchase snacks and other food items at local grocery stores as they wished, and had easy access to common foods and beverages throughout the day (cereal, bread, milk, yogurt, cheese, ham, biscuits, fruits, coffee, juice, nuts, sports bars, etc.) which they could consume *ad libitum*.

All the athletes received general information (20 min presentation) about the physiological demands and dietary needs of altitude training. This is a standard procedure at each altitude training camp arranged by the Norwegian Olympic Sports Center. Thus, most of the study participants had received similar information on several previous occasions. Furthermore, as a standard procedure, participants with pre-altitude serum ferritin <30 ug/L (females) or <40 ug/L (males) (*n* = 4, 2 in each group) were asked to take 90 mg elemental iron (ferrous fumarate) daily while at altitude. Participants were also asked to register daily body weight (nude/minimal clothing) first thing in the morning throughout the training camp and make adjustments to energy intake to maintain stable body weight if necessary. Study participants were not allowed to use any antioxidant supplements during the study but could use other types of dietary supplements and sports products as they wished. None of the athletes received individual dietary counseling during the study.

### The Dietary Intervention; In-Between Meal Snacks

The snacks provided for the in-between meals, both for the antioxidant and control group were provided by the Norwegian Olympic Sports Center, shipped to Spain from Norway, weighed with 1 g accuracy (Electronic kitchen scale, Page Evolution, Soehnle, Germany), packed and delivered daily at the same time for the whole 3-week period. The daily antioxidant-rich in-between meal snacks consisted of 750 ml fruit-berry-vegetable smoothie (3 beverages of 250 ml, rotating with four flavors), 40 g walnuts, 50 g dried berries and fruits, and 40 g dark chocolate (>70% cacao), while control in-between meals consisted of 500 ml milkshake, 330 ml recovery beverage, 50 g salt and sweat crackers, and 50 g white chocolate, as described in more detail previously (Koivisto et al., 2018). Selection of food items for the antioxidant group was done by experienced registered dietitians familiar with the population in collaboration with experts in the field of dietary antioxidants and is described elsewhere (Koivisto et al., 2018). Each participant received the same snack every day and they were asked to consume these snacks between their main meals, thus replacing some of their usual in-between meals. The compliance to the intervention was 95%, as previously reported (Koivisto et al., 2018). The compliance was controlled daily by a registered dietitian when subjects returned their food bags for refill. The total antioxidant content of both intervention and control foods was measured with the ferric reducing ability of plasma (FRAP) method, as previously depicted (Koivisto et al., 2019). The nutritional content of the daily antioxidant and control snacks, analyzed with a web-based dietary analysis program (see Analysis of the 24-h Recalls), can be found in Table 1. All participants received comprehensive oral and written information about the study, but the group allocation was not revealed to them. Whether the participants could correctly identify their group assignment was not assessed.

**Table 1:** Nutritional content of the daily antioxidant and control foods.

	**Antioxidant group**	**Control group**
Energy (kcal)	1,015	1,076
Carbohydrate (g)	138	136
Protein (g)	16.6	36.7
Fiber (g)	20.3	4.7
Fat (g)	39.7	40.9
SFA (g)	9.1	19.3
MUFA (g)	7.1	12.9
PUFA (g)	20.2	2.6
ω3 PUFA (g)	3.9	0.1
ω6 PUFA (g)	16.0	2.4
Vitamin A (RAE)	21	45
Retinol (μg)	2	41
Beta-carotene (μg)	277	62
Vitamin D (μg)	0.0	0.0
Vitamin E (α-TE)	5.3	2.7
Thiamin (mg)	0.5	0.3
Riboflavin (mg)	0.4	0.7
Niacin (mg)	5.3	1.8
Vitamin B6 (mg)	1.8	0.4
Folic acid (μg)	206	33
Vitamin B12 (μg)	0.1	1.4
Vitamin C (mg)	143	0
Calsium (mg)	182	487
Iron (mg)	6.2	1.8
Sodium (mg)	23	806
Potassium (mg)	2,879	819
Magnesium (mg)	299	81
Zink (mg)	2.7	2.3
Selenium (μg)	1	5
Copper (mg)	1.5	0.3
Phosphorus (mg)	447	543
Iodine (μg)	17.5	31.8

*MUFA, monounsaturated fatty acids; PUFA, poly-unsaturated fatty acid; SFA, saturated fatty acids*.

### Outcome Variables

The results of the primary outcome variables showed that the 3-week altitude camp resulted in significant increase in Hbmass (4.7%), and VO_2max_ (2.5%), but not swimming performance, with no difference between the groups (Koivisto et al., 2018). Furthermore intake of antioxidant-rich foods elevated the antioxidant capacity and attenuated some of the altitude-induced systemic inflammatory biomarkers in elite athletes with no impact on the altitude-induced oxidative stress or changes in acute cytokine responses to exercise stress-tests (Koivisto et al., 2019). The secondary outcome variables of the current study are described below.

#### Assesment of Dietary ntake

The dietary intake of participants was assessed with six interviewer-administered 24-h recalls on non-consecutive days; three times before the altitude training camp, and three times during the camp by an experienced registered dietitian (Figure 1). The menus provided by the training center (CAR) cafeteria was used in the 24-h recall setting in addition to household measures to assist in more precise estimates of portion sizes. Where an accurate weight of the food item was not available, estimates of portions sizes and/or weight per unit were used (e.g., mean weight of a specific fruit) listed in the Norwegian Food Composition Table (http://www.matvaretabellen.no/). The repeated 24-h recall was chosen as the dietary assessment method to minimize respondent burden, limit underreporting, while simultaneously collecting as accurate data as possible in this special population by using a highly trained interviewer (Larson-Meyer et al., 2018). In addition, as a measure of standardization, the dietary intake was recorded 24 h prior to collection of fasted blood samples before the attitude camp and the participants were asked to replicate it prior to the post altitude blood sampling.

##### Analysis of the 24-h recalls

To analyze the 24-h recalls of the study subjects, we utilized a web-based dietary analysis program (www.Kostholdsplanleggeren.no) that is based on the Norwegian Food Composition Table (http://www.matvaretabellen.no/). When a specific food item or a product (e.g., sports food products) was not found in the database we collected all available nutritional information about the product online or directly from the producer and inserted these data into the nutrition database of the program. Recipes of dishes were also added to the database with specific data for each food item (type and amount) in the dish. The intake of antioxidant-rich foods was calculated from the 24-h recalls and based on the major dietary contributors of antioxidants (Carlsen et al., 2010) included in the following food groups; berries, fruits, vegetables (except vegetables as an ingredient in sauces), juice, dark chocolate (>70% cocoa content), nuts, seeds, and coffee.

#### Blood Analysis

Blood samples were collected at four separate occasions; before, during (on day 5 and 18; only carotenoids) and after the 3-week altitude camp (Figure 1). All blood samples in the current study were collected after an overnight fast from an antecubital vein into two EDTA treated tubes and one serum separation tube (BD Vacutainer®). The measured blood parameters [serum iron, hemoglobin (hb), ferritin, reticulocyte hemoglobin (ReHb), magnesium, 25-OH-Dvitamin, vitamin E, folic acid, vitamin B12, low density lipoprotein cholesterol (LDL-c), high density lipoprotein cholesterol (HDL-c), sex hormone binding globulin (SHBG), testosterone and oestradiol] except for carotenoids, are part of the general assessment of the nutritional status of the athletes at the Norwegian Olympic Sports Center, and were analyzed by an accredited laboratory on the sampling day (Fürst laboratory, Oslo, Norway).

##### Analysis of carotenoids

Carotenoids were determined in plasma by HPLC using a modified version of a previously described method by Bastani et al. (2012). For analysis of carotenoids (lutein, zeaxanthin, β-cryptoxanthin, α-carotene, β-carotene, and lycopene) in plasma, HPLC-UV (high performance liquid chromatography with ultraviolet) detection was used. Proteins in plasma samples were precipitated by the addition of a 4.5 times volume of isopropanol containing 10 μg/mL trans-β-Apo-8′-carotenal (sigma-Aldich) (internal standard). Samples were mixed for 10 min and then centrifuged (3,000 g at 4°C) for 15 min. Aliquots of 20 μL of the supernatant were injected into the HPLC system. Plasma calibrators and controls were quantified against the standardized reference material 968c SRM from the National Institute of Standards and Technology (NIST).

### Statistical Analysis

Statistical analyses were performed using IBM SPSS Statistics 24.0. Normally distributed data are presented as mean ± standard deviation (SD), non-normally distributed data as median and range, and categorical data as ranks and percentages. Paired tests, either parametric or non-parametric, depending on data distribution, were used to determine whether there were significant effects of altitude training for the total study population. Two samples *T*-tests were performed to determine differences between the groups with regards to change in the parameters measured. If the data was non-normally distributed, a Wilcoxon-Mann Whitney *U*-Test was used. Changes in the number of users of supplements and sports products were analyzed using McNemar test. The groups were then compared based on the number of participants who either stopped, continued or started using sports products/supplements with Fisher exact test. The “qplot” function in the R (R for statistical computing, Vienna, Austria, Version 3.6.1.) -package “ggplot2” was used to plot the individual changes in carotenoids over time and the function “geom smooth” was applied to add a smoothed conditional mean for each group. Mixed model analysis was performed to determine whether there was a different longitudinal effect between the groups for carotenoids, which was measured at 4 time-points during the study. *P*-values are presented as nominal values. Bonferroni correction for multiple testing was not performed because the variables tested were not independent.

The magnitude of the differences between the groups, i.e., effect size, were determined using Cohen's d test. For non-parametric data, the effect size (r) of the intervention was calculated according to Rosenthal and Rosnow (1984). The effect size based on d or r, was interpreted according to Cohen's D (Rosenthal and Rosnow, 1984; Cohen, 1988).

#### Sample Size

As previously described (Koivisto et al., 2018) hbmass was used as the main variable for sample size estimation to detect a difference in change over time between the intervention and control group (Wehrlin et al., 2006; Gore et al., 2013). *Post-hoc* power analysis performed using an online power sample calculator (www.powerandsamplesize.com) with 1-Sample, 2-Sided Equality, 5% Type I error rate and 80% power deemed the current study to have adequate power (*n* = 7) to detect a significant change in energy intake from pre-altitude to altitude for the total study group based on an estimated increase in daily energy intake of 640 kcal as previously reported (Sanz-Quinto et al., 2019), and standard deviation for change in energy intake set at 600 kcal from our previous unpublished pilot study. Based on the 2-Sample, 2-Sided Equality test and similar data input we also have sufficient power to detect a difference in energy change between the intervention group and control group with 7 participants required in each group.

## Results

### Description of the Study Subjects

The cohort consisted of 23 males and 8 female Norwegian elite athletes, with a mean age of 23 years from five different summer sports: swimming (*n* = 11), rowing (*n* = 14) and kayaking, triathlon and middle-distance running (*n* = 6). More than half of the athletes had previous altitude training experience, as depicted in Table 2. Prior to the altitude training camp, in the start-up of their general preparation phase, the study subjects trained on average 19 ± 6 h per week, and the training load (number of hours) increased in both groups in similar manner, by 35% as previously described (Koivisto et al., 2018). There were no differences between the antioxidant and control group in any baseline characteristics presented in Table 2, or in any other baseline characteristics as reported previously (Koivisto et al., 2018, 2019).

**Table 2:** Baseline description of the study subjects.

	**All**	**Antioxidant group**	**Control group**	
	**(*n* = 31)**	**(*n* = 16)**	**(*n* = 15)**	***p***
Age (yrs)	23 ± 5	23 ± 5	24 ± 5	0.62
Height (cm)	185 ± 8	185 ± 8	185 ± 9	0.85
Weight (kg)	80.0 (38.8)	81.8 (31.8)	75.9 (38.8)	0.89
Fat percentage (%)	16.1 (18.2)	16.4 (12.4)	14.4 (18.2)	0.65
VO_2max_ (mL/kg/min)	67.3 (24.2)	67.6 (24.2)	66.5 (8.1)	0.43
Training volume (h per week)	18.7± 5.9	20.1 ± 6.3	17.0 ± 5.0	0.23
Sex				
Males	23 (74%)	12 (75%)	11 (73%)	1.0^b^
Females	8 (26%)	4 (25%)	4 (27%)	
Able-bodied/paralympic athletes				
Able-bodied athletes	27 (87%)	14 (88%)	13 (87%)	1.0^b^
Paralympic^a^	4 (13%)	2 (13%)	2 (13%)	
Previous altitude training experience				
Yes	21 (68%)	12 (75%)	9 (60%)	0.46^b^
No	10 (32%)	4 (25%)	6 (40%)	

*Values are presented as mean ± std or median (range) for non-normally distributed data or count (%). Values are presented as mean ± std or median (range) for non-normally distributed data or count. P-value indicates difference between the groups*.

b *Fisher's test*.

a *Two of the Paralympic athletes have spinal cord injury (paraplegy) and two have cerebral paresis, one in each group*.

### Dietary Intake

Analysis of the repeated 24-h recalls revealed that the number of daily meals consumed by the study subjects increased from 5.9 ± 1.0 pre-altitude to 6.5 ± 0.8 meals per day at altitude (*p* < 0.001). The change in meal frequency was not significantly different between the antioxidant and control group (*p* = 0.13).

#### Energy and Macronutrient Intake

##### Adjustments in dietary intake following ascent to altitude

**Figure 2:**
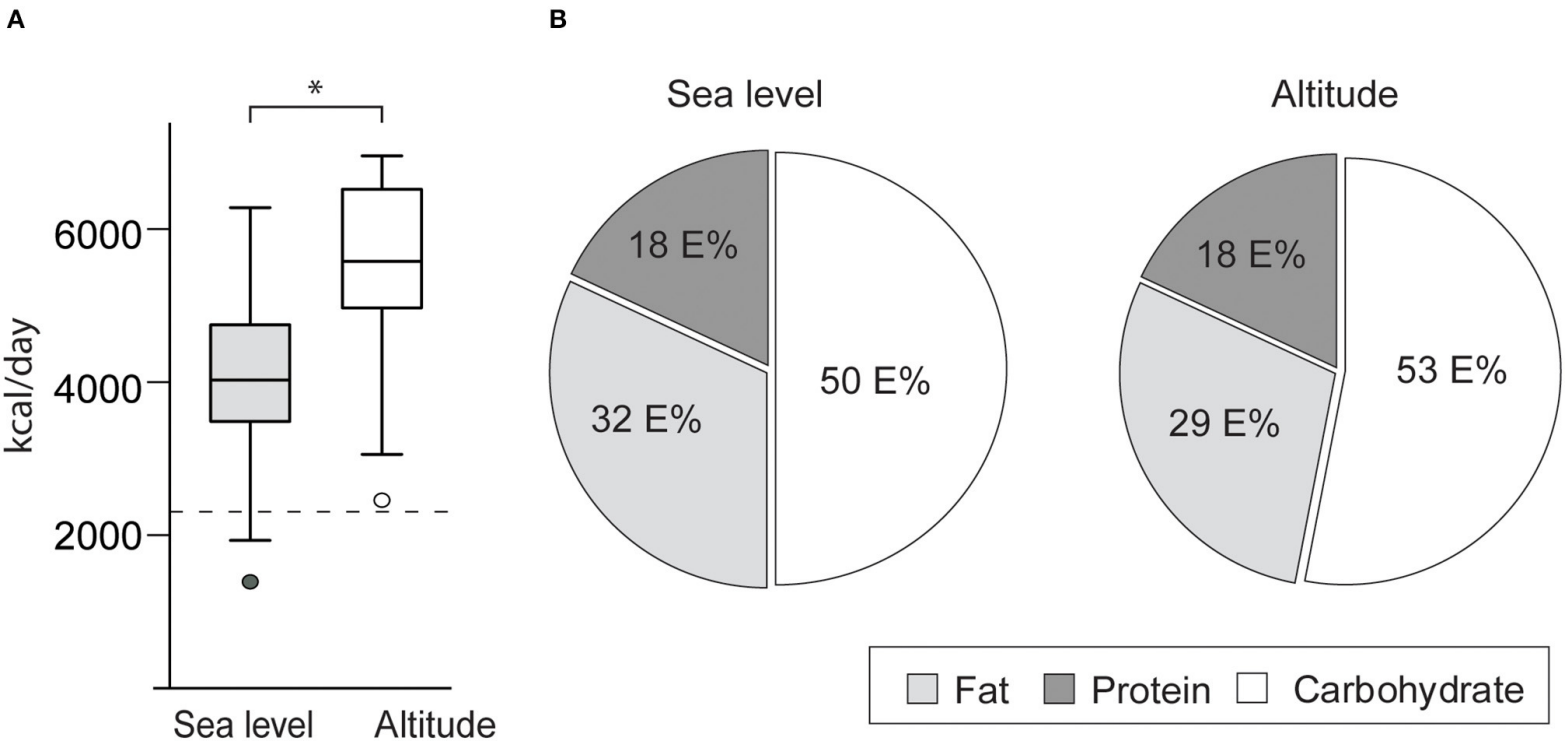
Mean daily energy intake pre-altitude and at altitude (2,320 m) in the whole population, with a dotted reference line to a healthy (non-athlete) Norwegian population (Totland et al., 2012) **(A)**, and mean energy contribution from carbohydrate, protein, and fat of the athletes' diets pre-altitude and at altitude **(B)**. ^*^indicates significant difference, *p* < 0.001. *P*-value is obtained from paired *t*-test.

Mean daily energy intake increased at altitude by 35% in the whole population, *p* < 0.001 (Table 3). Thirteen (42%) of the study participants increased their mean daily energy intake at altitude by more than 1,500 kcal. On average, the snacks provided to the study participants (1,000 kcal/day) accounted for 70% of the increase in daily energy intake. Energy contribution from carbohydrates increased most at altitude and accordingly changed the macronutrient composition of the athletes' diets, as shown in Figure 2. In the whole population there were significant increases in total carbohydrate intake (44% *p* < 0.001), including starch (26% *p* < 0.001), mono-and disaccharides (50% < 0.001), added sugar (48% *p* = 0.03) and fiber (30% *p* ≤ 0.001), as well as carbohydrate intake adjusted for body weight [g carbohydrates per kg body weight (BW)] (43% *p* < 0.001). Also, carbohydrate intake during exercise (g/h) more than doubled (+106%, *p* = 0.004) during the altitude training camp in the whole population.

**Table 3:** Mean daily energy, fluid, and macronutrient intake before (Sea level) and during (Altitude) the 3-week training camp at 2,320 m.

	**All**			**Antioxidant group**		**Controls**			
	**(*****n*** **=** **31)**			**(*****n*** **=** **16)**		**(*****n*** **=** **15)**			
	**Sea level**	**Altitude**	***p*_**paired**_**	**Sea level**	**Altitude**	**Sea level**	**Post altitude**	***p*_**change**_**	**ES_**change**_**
Energy (MJ)	16.9 ± 1.2	22.5 ± 6.0	<0.001^*^	17.4 ± 3.4	22.3 ± 5.9	16.4 ± 5.9	22.8 ± 6.3	0.23	0.4
Energy (kcal)	4,032 ± 1,133	5,462 ± 1,192	<0.001^*^	4,151 ± 814	5,497 ± 879	3,906 ± 1,417	5,426 ± 1,488	0.46	0.3
Fluid (L)	4.7 ± 1.2	6.5 ± 1.4	<0.001^*^	5.0 ± 1.2	6.5 ± 1.3	4.4 ± 1.2	6.5 ± 1.4	0.07	0.7
Carbohydrate (g)	519 ± 151	749 ± 176	<0.001^*^	544 ± 123	762 ± 142	492 ± 176	735 ± 211	0.71	0.8
Carbohydrate (g/kg BW)	6.5 ± 1.8	9.3 ± 2.1	<0.001^*^	6.8 ± 1.7	9.6 ± 2.1	6.2 ± 2.0	9.1 ± 2.2	0.67	0.2
Carbohydrate intake during	11.3 (53.0)	23.3 (44.0)	0.004^*^^W^	15 (53)	26 (44)	11.3 (36)	19 (40)	0.94^*MW*^	<0.1^r^
exercise >90 min (g/h)									
Starch (g)	230 ± 77	290 ± 77	<0.001^*^	240 ± 71	286 ± 61	233 ± 85	294 ± 98	0.36	0.3
Mono- and di-saccharides (g)	206 ± 82	309 ± 87	<0.001^*^	220 ± 78	329 ± 83	191 ± 86	287 ± 89	0.60	0.2
Added sugar (g)	63 (230)	93 (230)	0.030^*^^W^	59 (222)	73 (209)	68 (164)	117 (210)	0.37^*MW*^	0.2^r^
Fiber (g)	43 ± 12	56 ± 13	<0.001^*^	44 ± 10	63 ± 13	41 ± 14	49 ± 9	0.004^*^	1
Protein (g)	169 (202)	252 (171)	<0.001^*^^W^	180 (116)	231 (167)	162 (202)	276 (167)	0.018^*^^MW^	0.4^r^
Protein (g/kg BW)	2.2 (2.0)	3.1 (3.0)	<0.001^*^^W^	2.2 (1.0)	2.9 (1)	2.1 (2)	3.3 (3)	0.004^*^^MW^	0.5^r^
Fat (g)	146 ± 48	178 ± 45	<0.001^*^	147 ± 35	182 ± 36	145 ± 60	174 ± 54	0.66	0.2
SFA (g)	53 ± 21	70 ± 20	<0.001^*^	54 ± 17	67 ± 16	52 ± 24	72 ± 24	0.32	0.4
MUFA (g)	51 ± 19	56 ± 16	0.051	50 ± 16	55 ± 13	52 ± 24	57 ± 20	0.09	0.1
PUFA (g)	22 ± 8	30 ± 10	0.001^*^	22 ± 6	37 ± 7	22 ± 10	22 ± 6	<0.001^*^	0.8
Trans fat (g)	1.2 (2.5)	1.5 (77.4)	0.015^*^^W^	1.3 (2)	1.5 (2)	0.9 (3)	2 (77)	0.09^*MW*^	0.3^r^
ω3 PUFA (g)	5.2 ± 3	5.1 ± 2.5	0.95	5.4 ± 2.9	6.8 ± 1.9	4.9 ± 3.2	3.3 ± 1.5	0.011^*^	0.9
ω6 PUFA (g)	16 (27)	24 (30)	0.001^*^^W^	16 (18)	30 (25)	15 (27)	18 (14)	<0.001^*^^MW^	1.5^r^
Cholesterol (g)	535 ± 231	632 ± 197	0.048^*^	570 ± 230	628 ± 218	494 ± 235	636 ±180	0.40	0.3
Alcohol (g)	0.7 (30.0)	0.6 (15.0)	0.031^*^^W^	0.7 (15)	0.0 (15)	0.7 (30)	0.6 (1)	0.42^*MW*^	0.1^r^

*Values are presented as mean ± std or median (range) for non-normally distributed data*.

* *indicates significant difference, p < 0.05. The p-value, p_paired_ is obtained from paired tests testing the change from sea level to altitude for the total population (All) either by paired t-test or Wilcoxon (W) test. The p-value, pchange is obtained from comparing the change (from Pre-altitude to Altitude) between the groups using t-test or Wilcoxon-MW tests depending on the normality of the data. ES_change_ = effect size for change expressed as Cohen's d for parametric tests or Rosenthals (r) for non-parametric tests. SFA, saturated fat; MUFA, monounsaturated fat; PUFA, polyunsaturated fat; BW, body weight; MUFA, monounsaturated fat; PUFA, polyunsaturated fat; SFA, saturated fat*.

Dietary intake of most of the other macronutrients increased in the whole population as well, except for MUFA and ω3 PUFA that remained unchanged, and alcohol, which decreased at altitude (Table 3). When macronutrient intake was adjusted for energy intake, only alcohol and ω3 PUFA intake decreased at altitude (*p* = 0.023) while daily intake of all other nutrients remained unchanged (Supplemental Table 1).

**Figure 3:**
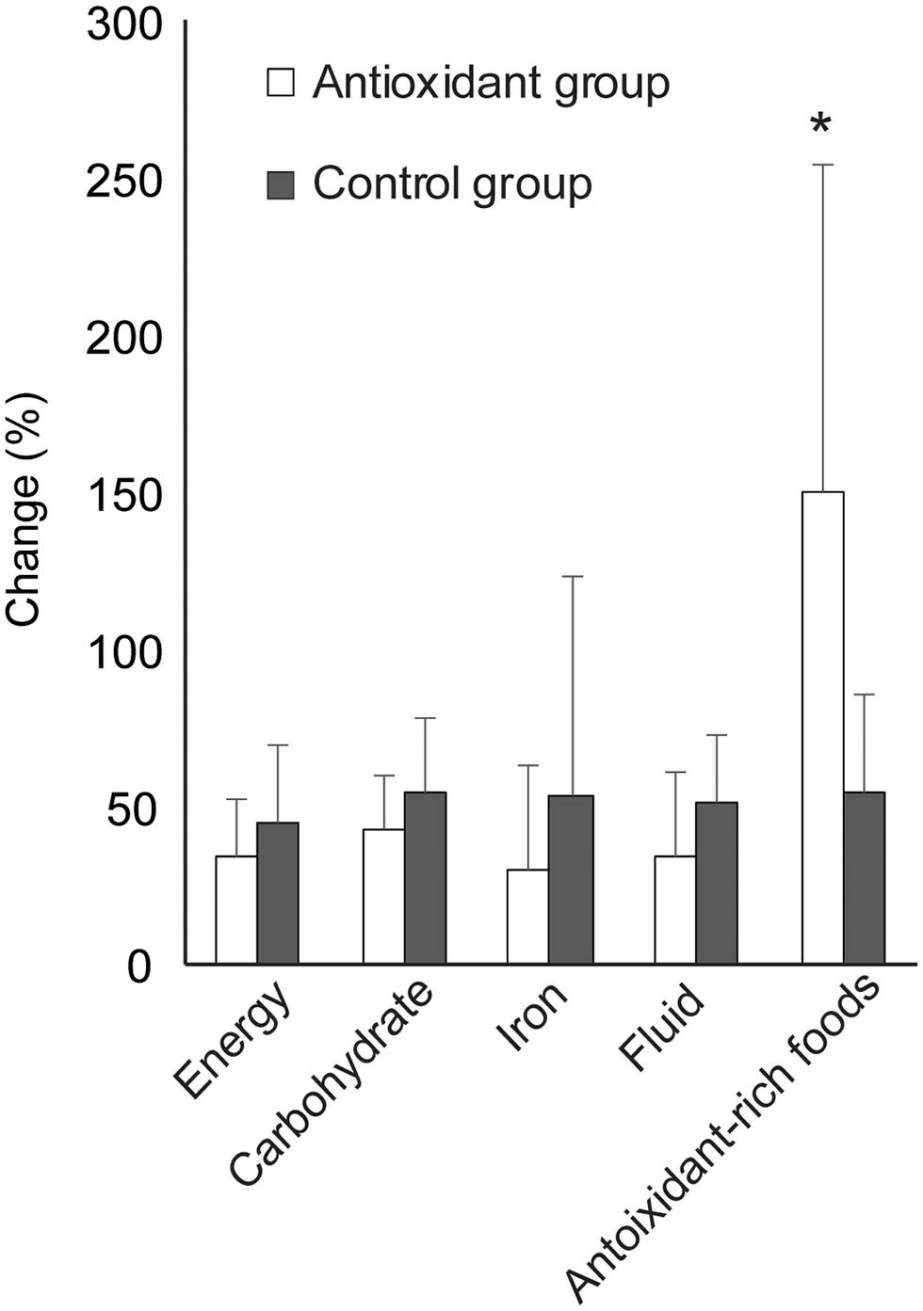
Relative change in the mean daily intake of principal nutrients for athletes training at altitude; energy, carbohydrate, iron, fluid, and antioxidant-rich foods in the antioxidant (*n* = 16) and control group (*n* = 15). Presented as mean altitude-sea level intra-individual change (%) and standard deviation. ^*^indicates significant difference, *p* < 0.05. *P*-value is obtained from paired *t*-test.

Furthermore, the daily intake of the key nutrients with presumably augmented requirements in athletes at altitude; energy, carbohydrate, fluid, iron, and antioxidants increased significantly in the whole population (*p* < 0.001, Table 3). Notably, only intake of antioxidant-rich foods increased significantly more in the intervention group as compared to controls (Figure 3).

##### Impact of the antioxidant-rich snacks on macronutrient intake at altitude

**Figure 4:**
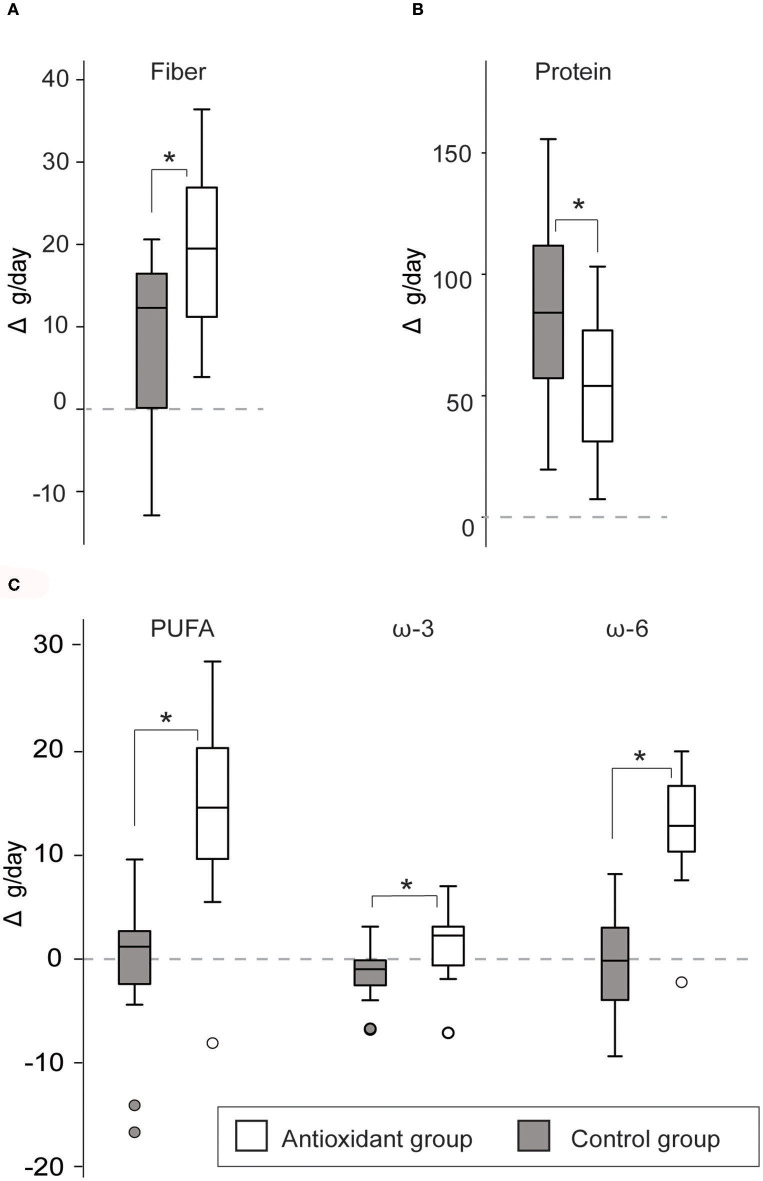
Change in the mean daily intake of macronutrients [fiber **(A)**, protein **(B)**, polyunsaturated fatty acids (PUFA), ω3 PUFA, and ω6 PUFA **(C)**] from Sea level to Altitude in the antioxidant and control group. Presented as mean Sea level—Altitude intra-individual change (%) and standard deviation. ^*^indicates significant difference, *p* < 0.05. The *p*-value is obtained from comparing the change from Sea level to Altitude between the groups using *t*-test or MW tests depending on the normality of the data.

There was a significantly larger increase in fiber [Effect size (ES) =1.0, *p* = 0.004], PUFA (ES = 0.8, *p* < 0.001), ω3 PUFA (ES=0.9, *p* = 0.011), and ω6 PUFA (ES = 1.5, *p* < 0.001) intake following ascend to altitude in the antioxidant group as compared to the control group, while the control group had a larger increase in protein intake as compared to the antioxidant group (ES = 0.4, *p* = 0.018) (Figure 4). See Table 3 for details of dietary macronutrient intake in antioxidant and control group before and during the altitude camp.

#### Micronutrient Intake

##### Adjustments in dietary micronutrient intake following ascent to altitude

**Figure 5:**
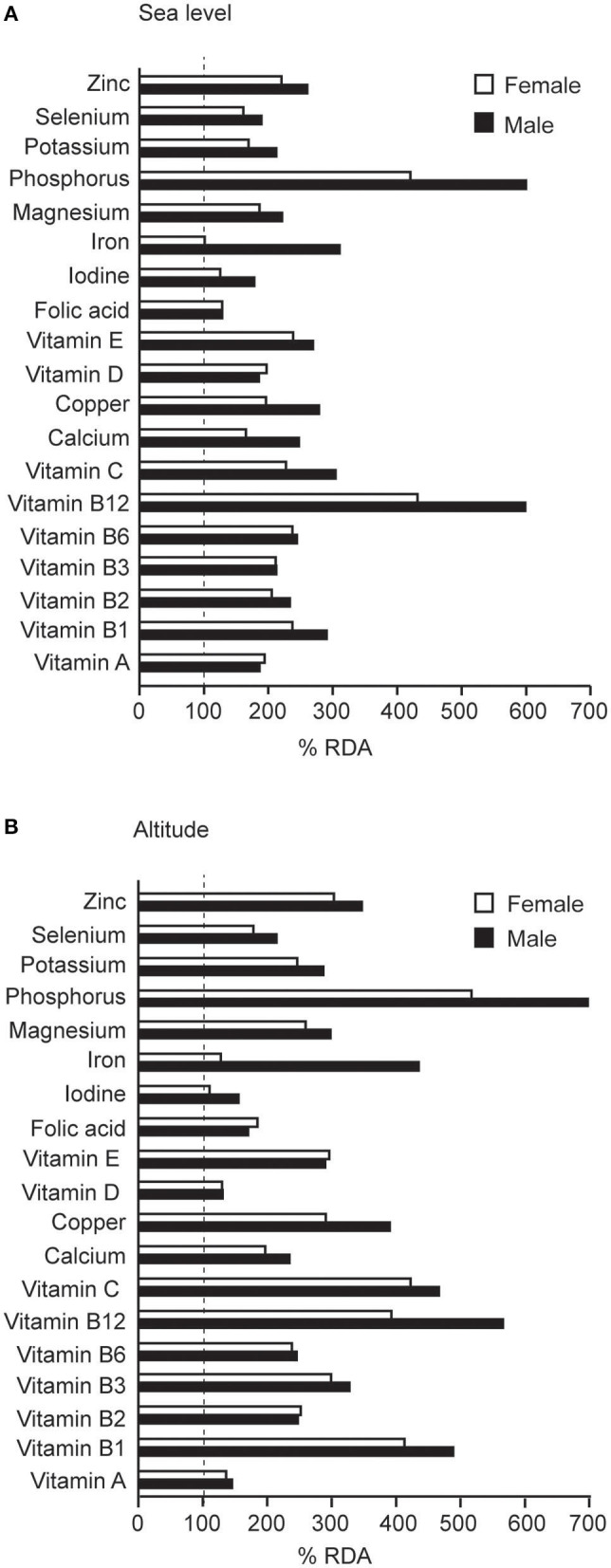
Mean daily intake of micronutrients before (sea level) **(A)** and during (altitude) **(B)** the 3-week altitude training camp as compared to recommended daily allowances (RDA) (dotted line) (Ministers NCo, 2012).

Mean daily intake of all micronutrients, except for vitamin B12, calcium, selenium and iodine, increased significantly (Table 4). There was a trend for an increase in β-carotene intake in the whole population (*p* = 0.06). Although when adjusted for total energy intake only vitamin B1, vitamin B6, and sodium intake increased at altitude, while the intake of vitamin A, vitamin D, β-carotene, vitamin B12, calcium, iodine, and retinol decreased at altitude (Supplemental Table 1). Intake of all micronutrients exceeded the recommended dietary allowances (RDA) of the Nordic countries (NNR) (Ministers NCo, 2012) both before and during the altitude training camp, as illustrated in Figure 5.

**Table 4:** Mean daily micronutrient intake before (Sea level) and during (Altitude) the 3-week training camp at 2,320 m.

	**All**			**Antioxidant group**		**Controls**			
	**(*****n*** **=** **31)**			**(*****n*** **=** **16)**		**(*****n*** **=** **15)**			
	**Sea level**	**Altitude**	***p*_**paired**_**	**Sea level**	**Altitude**	**Sea level**	**Altitude**	***p*_**change**_**	**ES_**change**_**
Vitamin A (RAE)	1,424 (4218)	1,227 (1,849)	0.035^*^^W^	1,654 (3,202)	1,262 (1,849)	1,194 (4,218)	1,083 (1,160)	0.24^MW^	0.2^r^
Retinol (μg)	898 (4097)	711 (1,679)	0.024^*^^W^	1,036 (2,545)	722 (1,647)	703 (4,097)	711 (1,095)	0.11^MW^	0.3^r^
β-carotene (μg)	3,808 (11,499)	4,560 (6,818)	0.06^W^	3,890 (1,094)	4,707 (6,818)	3,218 (6,772)	4,111 (6,092)	0.22^MW^	0.2^r^
Vitamin D (μg)	12.4 (56.0)	7.7 (35.0)	0.012^*^^W^	17.6 (45.0)	8.5 (35.0)	11.4 (54)	7.5 (30.0)	0.78*^*MW*^*	<0.1^r^
Vitamin E (α-TE)	24.9 ± 10.5	27.5 ± 7.4	0.08	25.3 ± 9.7	27.8 ± 4.8	24.4 ± 11.6	27.2 ± 9.6	0.92	<0.1
Thiamin (mg)	3.5 ± 1.1	5.9 ± 1.2	<0.001^*^	3.4 ± 0.8	5.9 ± 0.9	3.5 ± 1.4	5.8 ± 1.5	0.72	0.1
Riboflavin (mg)	3.4 ± 1.1	3.7 ± 0.8	<0.001^*^	3.4 ± 0.8	3.7 ± 1.0	349 ± 1.5	3.7 ± 0.7	0.94	<0.1
Niacin (mg)	37.5 ± 11.7	56.6 ± 11.1	<0.001^*^	38.4 ± 9.7	57.7 ± 10.3	36.5 ± 13.8	55.4 ± 12.2	0.93	<0.1
Vitamin B6 (mg)	3.4 ± 1.1	5.4 ± 1.2	<0.001^*^	3.6 ± 0.9	6.0 ± 1.1	3.2 ± 1.7	4.9 ± 1.0	0.10	0.6
Vitamin B12 (μg)	11.1 ± 5.0	10.4 ± 4.6	0.36	11.7 ± 3.1	10.1 ± 3.4	10.4 ± 5.8	10.7 ± 2.6	0.25	0.4
Folic acid (μg)	478 ± 147	643 ± 138	<0.001^*^	478 ± 103	729 ± 112	478 ± 187	551 ± 98	<0.001^*^	1.2
Vitamin C (mg)	213 ± 136	340 ± 109	<0.001^*^	212 ± 100	400 ± 99	214 ± 170	275 ± 82	0.024^*^	0.8
Iron (mg)	19.0 (77.0)	25.9 (135.0)	<0.001^*^^W^	20 (72)	27 (72)	18 (75)	24 (135)	0.86*^*MW*^*	<0.1^r^
Calcium (mg)	1,805 ± 671	1,784 ± 511	0.86	1,843 ± 510	1,637 ± 568	1,766 ± 826	1,941 ± 403	0.12	0.6
Sodium (mg)	4,149 ± 1,665	7,228 ± 2,535	<0.001^*^	3,853 ± 1,537	7,095 ± 2,254	4,465 ± 1,788	7,370 ± 2,879	0.68	0.2
Potassium (mg)	6,845 ± 1,888	9,359 ± 1,906	<0.001^*^	7,123 ± 1,492	10,160 ± 1,907	6,548 ± 2,251	8,505 ± 1,540	0.05	0.7
Magnesium (mg)	707 ± 228	956 ± 233	<0.001^*^	738 ± 192	1051 ± 234	674 ± 264	854 ± 189	0.06	0.7
Zinc (mg)	21.4 ± 6.4	28.4 ± 5.7	<0.001^*^	21.8 ± 4.5	28.1 ± 5.5	20.9 ± 8.1	28.8 ± 6.0	0.36	0.3
Selenium (μg)	104.8 ± 48.6	117.4 ± 28.3	0.14	102.1 ± 30.4	114.9 ± 27.2	107.8 ± 63.7	120 ± 30.2	0.97	<0.2
Iodine (μg)	229 (405)	183 (389)	0.24^W^	238 (263)	179 (383)	196 (384)	183 (205)	0.66*^*MW*^*	0.1^r^
Copper (mg)	2.3 ± 0.7	3.3 ± 0.7	<0.001^*^	2.5 ± 0.5	3.7 ± 0.6	2.3 ± 0.9	2.8 ± 0.6	<0.001^*^	1.2
Phohsphorus (mg)	3,298 ± 1,017	3,882 ± 753	<0.001^*^	3,396 ± 667	3,794 ± 782	3,194 ± 1,311	3,977 ± 736	0.17	0.5

*Values are presented as mean ± std or median (range) for non-normally distributed data*.

* *indicates significant difference, p < 0.05. The p-value, ppaired is obtained from paired tests testing the change from sea level to altitude for the total population (All) either by paired t-test or Wilcoxon (W) test. The p-value, pchange is obtained from comparing the change (from Pre-altitude to Altitude) between the groups using t-test or Wilcoxon-MW tests depending on the normality of the data. ES_change_ = effect size for change expressed as Cohen's d for parametric tests or Rosenthals (r) for non-parametric tests*.

##### Impact of the antioxidant-rich snacks on micronutrient intake at altitude

**Figure 6:**
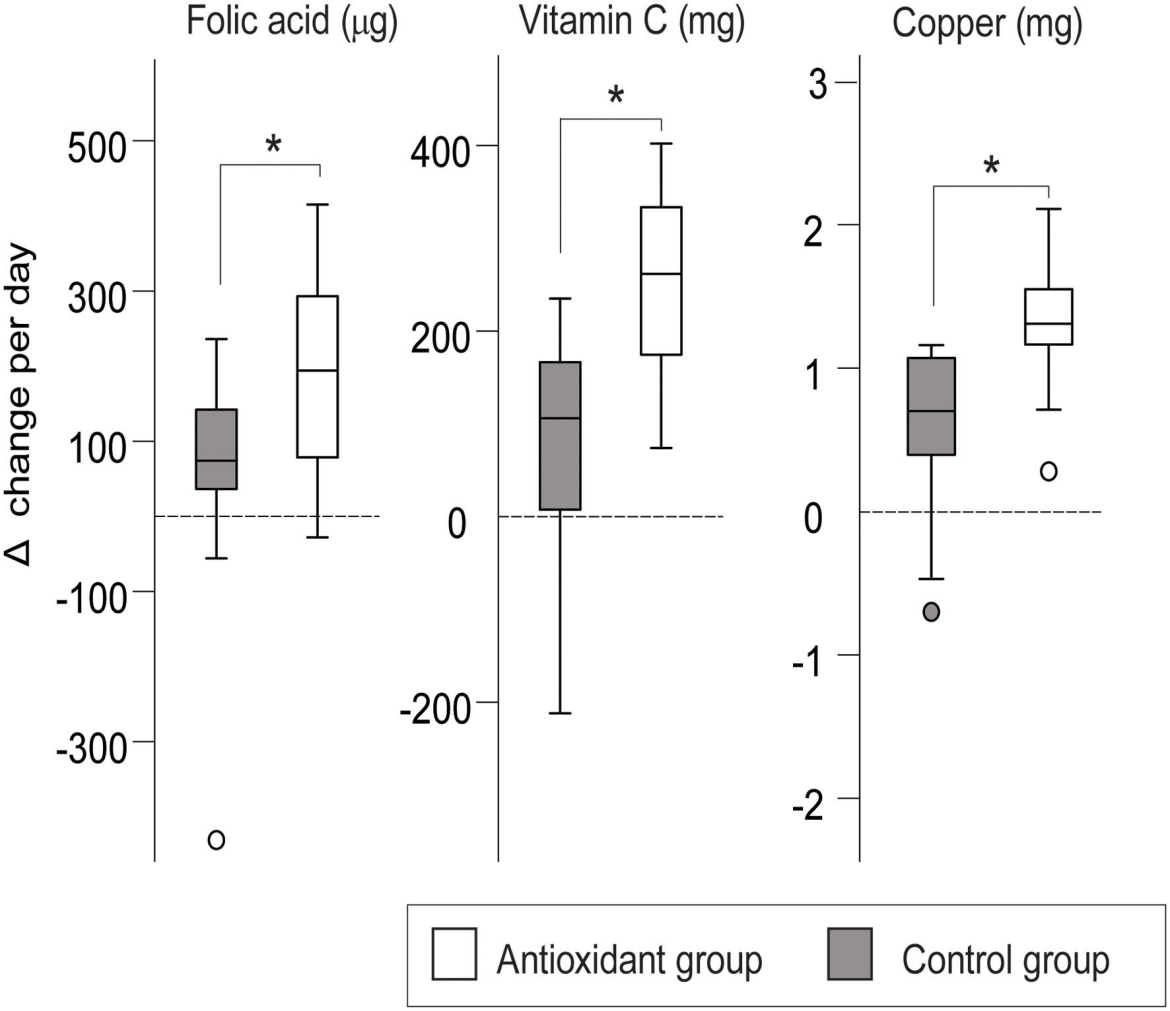
Change in the mean daily intake of micronutrients (folic acid, copper, vitamin C) from Sea level to Altitude in the antioxidant and control group. Presented as mean Sea level—Altitude intra-individual change (%) and standard deviation. ^*^indicates significant difference, *p* < 0.05. The *p*-value is obtained from comparing the change from Sea level to Altitude between the groups using *t*-test or MW tests depending on the normality of the data.

There was a larger increase in the daily intake of folic acid (ES = 1.2, *p* < 0.001), vitamin C (ES = 0.8, *p* = 0.024), and copper (ES = 1.2, *p* < 0.001) in the antioxidant group (Figure 6), as well as a trend for a larger increase in magnesium (ES = 0.7, *p* = 0.06) and potassium (ES = 0.7, *p* = 0.05) intake.

#### Use of Dietary Supplements and Sports Products

##### Adjustments in the use of dietary supplements and sports products at altitude

Twenty out of 31 study subjects (65%) used dietary supplements before the training camp, mostly ω3 PUFA, multi-vitamin, mineral-electrolyte, probiotic and vitamin D supplements. Most of the athletes who used dietary supplements (65%) consumed only one type of supplement, while three athletes used 3 types of supplements. The dietary supplement use did not change following ascend to altitude (*p* = 0.55). In contrast, the use of sports products (including any carbohydrate-electrolyte drink/powder, energy bars, energy gels, recovery powder, recovery beverage, protein powder, and protein bars used by the study participants) increased significantly at altitude (*p* < 0.001), where all study subjects reported using at least one type of sport product as compared to 61% at sea level. Sports drink (carbohydrate-electrolyte powder) was most used sports product followed by energy bar. The share of athletes using sports drinks increased from 25 to 77% at altitude (*p* < 0.001).

##### Impact of antioxidant-rich snacks on use of supplements and sports products

There was no significant impact of the intervention on the change in intake of dietary supplement or sports product at altitude except for the use of recovery beverage which increased more in the control group *p* < 0.001. This was expected due to the inclusion of a recovery beverage in the control diet.

### Nutrition-Related Blood Parameters

#### Impact of Altitude Training

The 3-week altitude training camp resulted in reduced s-iron and s-ferritin and an increase in hb (Koivisto et al., 2018) and ReHb in the whole population (Table 5), illustrating the heightened incorporation of iron into the new hemoglobin molecules. Despite an increase in dietary take of folic acid and vitamin B12 the serum concentration of these nutrients remained unchanged. Also, serum HDL-c and LDL-c remained unchanged throughout the study, while serum 25-OH-D3-vitamin decreased in the whole population. Plasma concentration of zeaxanthin, a carotenoid, increased over time (*p* = 0.01, fixed effect) peaking at visit 3 (day 18) at altitude (β: 0.04, *p* < 0.001). Another carotenoid, β-cryptoxanthin, also increased significantly during the study peaking at visit 3 (day 18) at altitude (β: 0.08, *p* = 0.013) and 4 (β: 0.07, *p* = 0.031). The other measured carotenoids; lycopene, α- and β-carotene, did not change significantly over time.

**Table 5:** Blood parameters before (pre-altitude) and after (post-altitude) the 3-week altitude training camp.

	**All**			**Antioxidant group**		**Controls**		
	**(*****n*** **=** **31)**			**(*****n*** **=** **16)**		**(*****n*** **=** **15)**		
	**Pre-altitude**	**Post-altitude**	***p*_**paired**_**	**Pre-altitude**	**Post-altitude**	**Pre-altitude**	**Post-altitude**	***p*_**change**_**
s-25-OH-vit D (nmol/L)	112 (109)	96 (57)	<0.001^*^^W^	113.5 (109)	98.9 (58)	110.5 (36)	96.9 (41.0)	0.257
s-Vitamin E (pmol/L)	24.5 (24.0)	27.0 (30.0)	0.040^*^^W^	24.0 (17.0)	25.5 (25)	25.5 (20.0)	29.9 (23.0)	0.751
s-Folate (nmol/L)	18.0 (28.0)	19.0 (34.6)	0.569*^*W*^*	19.8 (28)	19.0 (23.4)	18.2 (21.0)	19.0 (34.6)	0.449
s-Vitamin B12 (pmol/L)	355 ± 141	351 ± 115	0.610	380 ± 180	359 ± 135	332 ± 93	344 ± 92	0.372
s-Magnesium (mmol/L)	0.81 ± 0.06	0.87 ± 0.32	0.362	0.79 ± 0.05	0.79 ± 0.05	0.83 ± 0.07	0.93 ± 0.40	0.394
s-ReHb (pg)	33.8 ± 1.2	34.4 ± 1.2	<0.001^*^	34.5 ± 1.0	35.0 ± 0.9	33.3 ± 1.0	33.8 ± 1.2	0.633
s-LDL-c (mmol/L)	2.6 ± 0.7	2.7 ± 0.8	0.380	2.5 ± 0.7	2.6 ± 0.9	2.6 ± 0.6	2.6 ± 0.7	0.572
s-HDL-c (mmol/L)	1.7 ± 0.4	1.6 ± 0.3	0.068	1.7 ± 0.4	1.6 ± 0.3	1.7 ± 0.4	1.5 ± 0.3	0.606
s-SHBG (nmol/L)	41.5 ± 17.3	42.3 ± 16.3	0.608	42.3 ± 15.5	45.3 ± 18.8	40.8 ± 19.5	39.9 ± 14.9	0.394
s-Testosterone (nmol/L)	14.3 ± 5.6	17.1 ± 6.2	0.052	16.0 ± 6.0	16.4 ± 5.9	13.0 ± 5.1	16.2 ± 4.3	0.134
(males, All *n* = 23, AO *n* = 12, CON *n* = 11)								
s-Oestradiol (nmol/L)	0.25 ± 0.09	0.28 ± 0.15	0.523	0.23 ± 0.11	0.29 ± 0.11	0.27 ± 0.08	0.28 ± 0.20	0.643
(females, All *n* = 8, AO *n* = 4, CON *n* = 4)								

*Values are presented as mean ± std or median (range) for non-normally distributed data*.

* *indicates significant difference, p < 0.05. The p-value, ppaired is obtained from paired tests testing the change from Pre-altitude to Post-altitude for the total population (All) either by paired t-test or Wilcoxon (W) test. The p-value, pchange is obtained from comparing the change (from Pre-altitude to Post-altitude) between the groups using t-test or Wilcoxon-MW tests depending on the normality of the data. AO, Antioxidant group; CON, Controls; HDL-c, high density lipoprotein cholesterol; LDL, low density lipoprotein cholesterol; ReHb, Reticulocyte hemoglobin*.

#### Impact of Antioxidant-Rich Snacks

**Figure 7:**
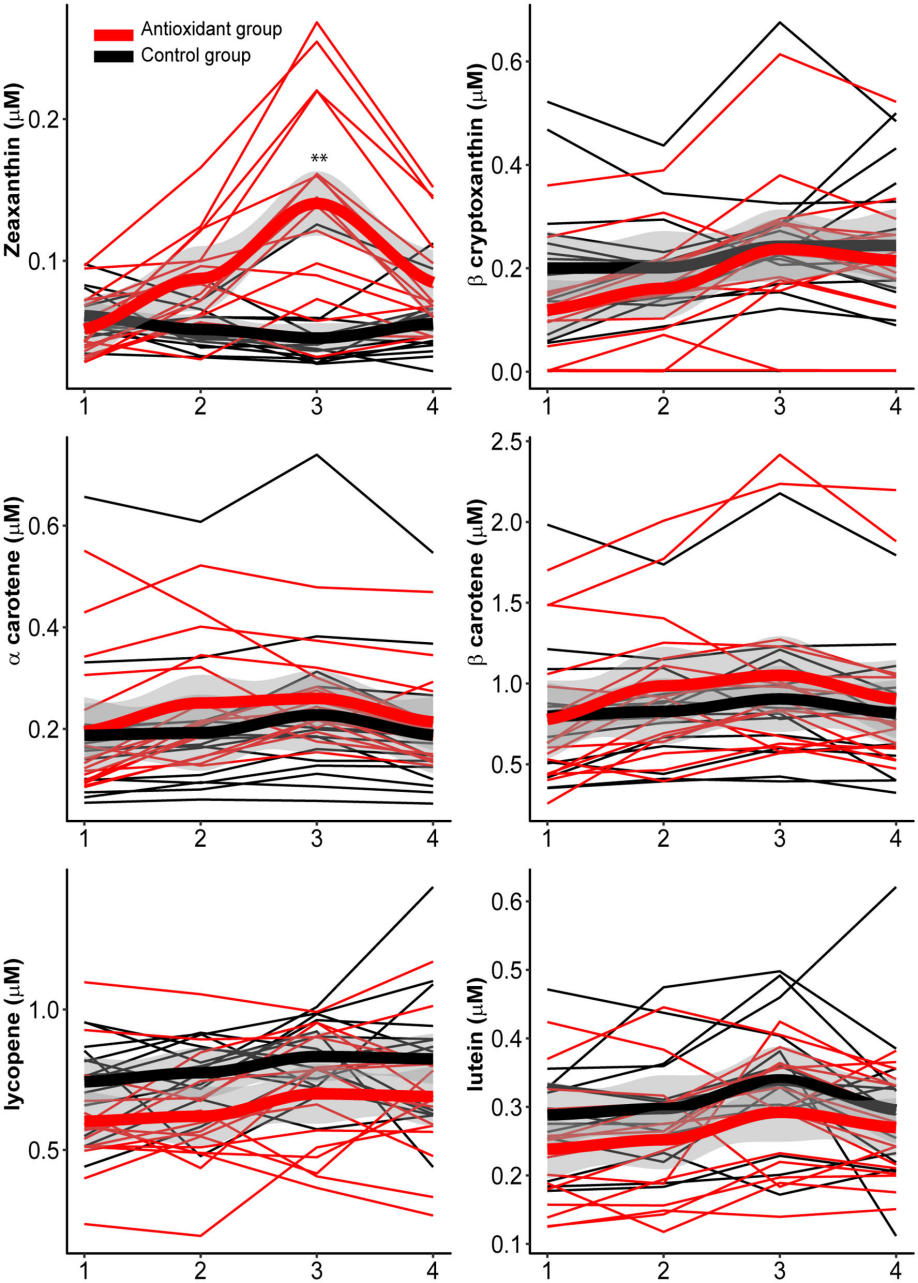
Carotenoid (zeaxanthin, β-cryptoxanthin, α-carotene, β-carotene, lycopene, and lutein) concentration pre-altitude (1), on day 5 (2), and 18 (3) at altitude (2,320 m), and post-altitude (4) in the antioxidant and control group. ^**^mixed model analyses indicate significant time x group effects for zeaxanthin. The “qplot” function in the R (R for statistical computing, Vienna, Austria, Version 3.6.1.)—packages “ggplot2” and the function “geom smooth”.

There was a significant difference in change over time in zeaxanthin between the groups (*p* < 0.001, fixed effect), with a larger increase in the antioxidant intervention as compared to controls, peaking at visit 3 (day 18) (β: 0.1, *p* < 0.001) (Figure 7), while the remaining carotenoids (β-cryptoxanthin, α-carotene, β-carotene, lycopene, and lutein) did not change differently between the groups. The change in the measured serum vitamin, mineral and hormone variables from pre-to post-altitude was not significantly different between the antioxidant and control group (Table 5).

#### Sex Differences in Dietary Intake and Blood Parameters

Although the study was not designed to investigate sex differences, we performed explorative analysis that showed that most of the dietary intake variables did not change differently between males and females. Only increase in mean daily energy (36% *p* = 0.009), vitamin B3 (22% *p* = 0.033), iron (33% *p* = 0.048), and sodium (80% *p* = 0.015) intake from sea level to altitude was significantly larger among males as compared to females (Supplemental Table 2). Changes in blood parameters following altitude camp were significantly different between males and females only for ferritin and iron. Ferritin reduced significantly more in the males −19.6% *p* = 0.049 while s-iron reduced significantly more among the females −44.5% *p* = 0.034. There was also a trend for an increase in s-testosterone during the altitude camp among male study subjects (19.5%, *p* = 0.05) (Supplemental Table 2).

## Discussion

The demanding environmental setting while training at altitude leaves little room for inadequate dietary routines for athletes who desire optimal physiological adaptation and sound health. Thus, investigating current dietary practices in this unique population is highly valuable. Moreover, although altitude-specific dietary recommendations for athletes exist, the impact or feasibility of them has not been investigated before. Herein we provide novel observations of dietary adjustments undertaken by elite endurance athletes during a 3-week training camp at moderate altitude (2,320 m).

### Dietary Adjustments at Altitude

During the 3-week training camp our study population managed to increase their dietary intake of energy, carbohydrate, fluid, iron and antioxidant-rich foods as recommended for athletes training at moderate altitude by IOC (Maughan and Burke, 2012). The energy intake in our cohort increased by 35% (5.6 MJ/day). Interestingly this increase matched well with the relative increase in training hours during the same period (35% increase). However, this synchronism does not necessarily reflect energy balance because the number of training hours accounts only for a small part of the total daily energy expenditure (TEE) (Westerterp, 2018). Several factors contribute to TEE and most likely both training intensity (Solli et al., 2017), non-exercise activity and RMR (Woods et al., 2017b) were altered during the altitude camp. Thus, no firm conclusions can be made about the changes in TEE or its underlying factors. Importantly though, all the athletes managed to maintain stable body weight and stable lean body mass, measured with dual energy x-ray absorptiometry, as previously described (Koivisto et al., 2018). In contrast to our results, a study with similar population of world class athletes training at 2,200 m reported a mismatch between *ad libitum* energy intake and energy expenditure (negative energy balance by 1.4 MJ/day, measured with doubly labeled water) over a period of seven days, resulting in a significant weight loss (Fudge et al., 2006). Other studies have also reported reduced energy intake (Westerterp et al., 2000; Fudge et al., 2006), compromised appetite (Karl et al., 2018) and increased resting energy expenditure (Woods et al., 2017b), all contributing to a significant loss of body mass (Kayser, 1992; Fudge et al., 2006). However, these studies were conducted at a higher altitude and with a slightly different population of athletes. We did not measure RMR or energy availability in our study. However, our cohort maintained stable body weight, increased their energy intake, obtained a larger than expected increase in hbmass (Koivisto et al., 2018) and demonstrated a strong trend for increase in s-testosterone [a biomarker for energy availability among male athletes (Mountjoy et al., 2018)]. Collectively these results suggest that energy availability was adequate during the altitude training camp, which is considered as one of the main nutritional goals for athletes managing a successful training camp at altitude (Stellingwerff et al., 2019). Also, the current study was conducted outside the competition and weight making period, and most of the athletes were experienced altitude users aware of the increased energy demands and potential downside of negative energy availability. Furthermore, easy access to varied foods/snacks probably also helped to overcome poor appetite in our cohort, which is a common side-effect of altitude exposure (Debevec, 2017).

The increased energy from daily carbohydrate intake (3.9 MJ/day) was the main contributor to the increase in total energy intake. Daily carbohydrate intake at altitude in our cohort was quite similar to other studies in elite endurance athletes at altitude (Christensen et al., 2002; Fudge et al., 2006; Beis et al., 2011), and well within the current recommendation for athletes (6–12 g/kg body weight), while protein intake was commensurately higher, and also above the recommended intake by American College of Sport medicine (Thomas et al., 2016). Carbohydrate intakes at the upper end of the recommendation scale (>9 g/kg body weight) are rarely reported among athletic populations, while sub-optimal carbohydrate intake is still more common (Wardenaar et al., 2017a; Jenner et al., 2019). Indeed, a large descriptive study of Dutch elite endurance athletes' dietary intake at sea level revealed sub-optimal carbohydrate intake in the light of current recommendations, but the inclusion of sport nutrition products in the analysis resulted in a somewhat lower prevalence of sub-optimal carbohydrate intakes (Wardenaar et al., 2017a). In the current study the carbohydrate consumption (sports products) during workouts lasting >90 min more than doubled [22 (Solli et al., 2017) g per hour], although still far below the current recommendations (30–90 g carbohydrate per h) (Thomas et al., 2016). As a comparison, while only 20% of Kenyan middle- and long-distance runners consumed commercially available sports drink at altitude (Beis et al., 2011), the incidence of *ad libitum* carbohydrate-electrolyte beverage use at altitude in our cohort was 100%. A recent meta-analysis (Griffiths et al., 2019) indicate that the reliance on carbohydrate oxidation at the same absolute training intensity might be slightly greater at altitude than at sea level (Young et al., 2019), thus the adjustment in carbohydrate intake in our cohort seems to be legitimate, also in the light of increased total training load. Of note, none of the study participants deliberately utilized training with low carbohydrate availability (Impey et al., 2018) while at altitude.

Both s-ferritin and s-iron decreased (Koivisto et al., 2018) despite a significant increase in dietary iron intake, suggesting that iron had to be mobilized from body stores to meet the demands of accelerated erythropoiesis at altitude. However, as we previously reported (Koivisto et al., 2018) the total hbmass increased significantly (4.7%) in response to altitude training, indicating no limitations in iron stores. In the current study we followed the 2012 IOC guidelines (Bergeron et al., 2012) that suggest iron supplementation for athletes with low pre-altitude ferritin (<30 nmol/L for females and <40 nmol/L for males), applying for four athletes in our cohort. In contrast, several recent studies recommend iron supplements for athletes at altitude regardless of normal s-ferritin to enhance the hbmass response (Govus et al., 2015; Garvican-Lewis et al., 2016a; Stellingwerff et al., 2019). Hence, the relative contribution of iron from different origins for the optimal hemoglobin mass increase requires further investigation.

The increased intake of fruits, vegetables, and berries in the whole population indicates that the athletes adjusted their diets to meet the recommendation to increase dietary sources of antioxidants (Maughan and Burke, 2012). However, plasma β-carotene remained unchanged. The discrepancy might be explained by the change in type of fruits and vegetables consumed by the athletes (larger intake of fruits and vegetables with lower β-carotene content, like broccoli and bananas), in addition to the dietary, hormonal, and genetic factors that affect the bioavailability and blood concentration of β-carotene (Moran et al., 2018).

Given the considerable increase in energy intake at altitude it was expected that the intake of most macro-and micronutrients increased. However, when dietary intake was adjusted for total energy intake most micronutrient intake remained unchanged or even decreased, reflecting change in dietary choices. RDA for all micronutrients was covered both before and during the altitude training camp, and accordingly most nutrition-related blood variables remained unchanged apart from a decrease in s-25-OH-D vitamin and the aforementioned s-iron. The observed reduction in s-25-OH-D vitamin might be explained by reduced access to vitamin-enriched spreads (source for vitamin A, D, retinol) and seafood, including mackerel spreads regularly consumed in Norway [source for vitamin D, iodine, omega-3 (Torris et al., 2018)] as well as lower dairy consumption [source for vitamin D, A, iodine, B12 (Rizzoli, 2014; Carlsen et al., 2018)]. Peculiarly, the increased exposure for UVB radiation in Spain vs. Norway combined with an increase in outdoor training contradicts this finding.

Intake of fluid, another key nutrient at altitude (Milledge, 1992), increased following ascend to altitude and the whole population maintained adequate fluid balance throughout the camp (Koivisto et al., 2018). This is in contrast to previous findings reporting lower fluid intake among athletes at altitude (Beis et al., 2011). This discrepancy might owe to the self-monitoring of hydration status. In the current study the participants were instructed to track their body weight daily (day to day changes reflecting hydration status) which facilitated fluid intake adjustments, if necessary. Also, the fluid from the daily antioxidant-rich (750 ml) and control (550 ml) snacks (contributing with 22 and 50% to the increase in total daily fluid intake, respectively) most likely aided to the maintenance of fluid balance.

### Impact of Antioxidant-Rich Snacks on the Dietary Adjustments at Altitude

A central new finding of the study revealed that the antioxidant-rich snacks not only increased the intake of antioxidants, but also improved the macronutrient and micronutrient composition of the athletes' diets. Among the macronutrients; PUFA, ω3 PUFA, and ω6 PUFA intake all increased in the intervention group as compared to controls. The recommended intake of ω3 PUFA (>1 E%) and PUFA (5–10 E%) at altitude was only achieved in the antioxidant group. This can be explained by the higher content of these nutrients in the intervention foods, especially in walnuts. Protein intake increased more in the control group, most likely because of the higher protein content of the control foods. Although the intervention and control snacks were matched for energy and carbohydrate content, the protein content was unfortunately higher in the control foods and lead to a higher increase in total protein intake in the control group (114 g/day) compared to the intervention group (51 g/day). However, the control snacks contributed only to 32% of the increase in the total daily protein intake, thus food choices independent of these snacks accounted for most of this difference. Importantly, no difference was found in changes in body weight or lean body mass (Koivisto et al., 2018). This suggests that the extra protein was merely used as an alternative source for energy.

Moreover, the antioxidant-rich food intervention elicited a larger increase in vitamin C, folic acid and copper intake, and a trend for higher magnesium and potassium intake as well, all in line with the finding that intake of vegetables, fruits, berries, and juice increased more in the intervention group as compared to controls. Dietary vitamin C intake in the intervention group exceeded the proposed vitamin C requirement for athletes at altitude (>200 mg per day) (Neubauer and Yfanti, 2015). Previous examinations of athletes' dietary intake at sea level have revealed lower vitamin C, copper and folic acid intake (Palazzetti et al., 2004; Wardenaar et al., 2017b). Presumably the larger total energy intake in the current study, in addition to the dietary intervention at altitude explains these differences.

Furthermore, we detected a significant increase in plasma zeaxanthin concentration in the antioxidant group. Zeaxanthin, which belongs to the carotenoid subgroup xanthophylls, is found in orange, yellow, and green vegetables and fruits like pepper, corn, carrot, apricot, spinach, kale, and broccoli (Abdel-Aal el et al., 2013). The intervention foods indeed contained carrots, apricot and other yellow-orange colored plant foods that might explain this finding. Yet, the change in the rest of the carotenoids was not different between the groups. A potential explanation could be that other phytochemicals like flavonoids, rather than carotenoids alone, were elevated, since the intervention was food-based and contains a myriad of phytochemicals.

### Limitations

It may be argued that we should have used weighed food records for dietary assessments as it is considered “gold standard.” However, weighed food records are prone to under-reporting, especially in high energy consumers such as endurance athletes because of the challenges with keeping a food record while consuming large portion sizes, consuming frequent meals, and using various sports products (Deakin et al., 2015). We chose repeated interviewer-administered 24-h recalls to reduce respondent burden, which may cause the individual to change their dietary behavior, and drop-out from the study (Capling et al., 2017). Furthermore, the interviewer used a multi-pass approach to assist the athletes with remembering all foods consumed, which is a well-known limitation with all retrospective dietary assessment methods, finishing the 24-recall with a review of data collected while probing for additional foods. Since dietary recalls have shown underreporting at the high intakes and overreporting at the low intakes (flat-slope syndrome) (Magkos and Yannakoulia, 2003), this might have reduced the spread in our data. Also, underreporting may progress at higher energy expenditures (Westerterp et al., 1986) and a recent systematic review showed that self-reported energy intake is underestimated by 19% among athletes (Capling et al., 2017), thus the observed adjustments in energy intake in the current study might be even larger in reality. Nevertheless, this potential misreporting would be similar between the groups and would not affect the group comparisons. Also, we did not measure energy expenditure, and can only elucidate on athletes' energy balance with indirect measures. Furthermore, we did not include a control group at sea level thus we cannot precisely define the contribution of hypoxic exposure alone on the dietary adjustments. Finally, although dining at a training center cafeteria is a common practice among athletes, both at home and during training camps, the food environment at altitude differs of that at home (sea level) and might have limited some food item choices. Whether the results of the study apply to less experienced athletes who must prepare all their meals themselves (no access to cafeteria or restaurants) remains to be investigated.

## Conclusions

In conclusion, this study provides novel information that experienced endurance athletes adjust their dietary intake while training at moderate altitude in an international training center setting to meet the altitude-specific dietary recommendations. The other principle finding of this study reveals that an intervention designed to increase antioxidant-rich food intake, provided as in-between meal snacks, not only increased intake of antioxidants but also improved the overall macro-and micronutrient composition of the athletes' diets, despite of accounting only for 18% of total daily energy intake. This information may be useful for sports dietitians, support staff, coaches, and athletes to increase the awareness about the substantial impact that snacks consumed in between meals have for the overall diet quality, and to motivate to make a conscious effort to have a variety of antioxidant-rich foods easily available for athletes utilizing altitude training.

## Data Availability Statement

The datasets presented in this article are not readily available because there is a risk of violating participant anonymity. However, apart from some minor restrictions, the majority of the raw data supporting the conclusions of this article will be made available upon request. We will provide full access to data underlying the main findings presented in the current article i.e., dietary intake, blood vitamin, mineral, hormone, and carotenoid concentrations with group allocation. The restrictions for data sharing apply to the descriptive information (Table 2) of the relatively small group of identifiable elite athletes with only a few athletes in some of the sport categories. Requests to access the datasets should be directed to sivb@nmbu.no.

## Ethics Statement

The studies involving human participants were reviewed and approved by The Norwegian Regional Ethics Committee (REK number 626539, https://rekportalen.no/). The patients/participants provided their written informed consent to participate in this study.

## Author Contributions

The study was conceived by AK-M and designed by AK-M, GP, IP, IG, TR, NB, RB, and SB. AK-M, IP, NB, GP, and SB conducted the research. AK-M was responsible for the dietary assessments and test logistics while NB did the plasma carotenoid analysis. RB and SB provided essential reagents or provided essential materials. AK-M and SB analyzed data, performed statistical analysis, wrote the paper, and had the primary responsibility for final content. All co-authors commented and approved the final version of the document.

## Conflict of Interest

RB who has shares in AS Vitas, Oslo, Norway. Some of the food items (smoothies, nuts, and dried berries) in the study were sponsored by Bama (Norwegian fruit, vegetable, and berry importer), while milkshakes and YT recovery beverages were sponsored by TINE (Norwegian dairy company). The remaining authors declare that the research was conducted in the absence of any commercial or financial relationships that could be construed as a potential conflict of interest.
