# Supplementary material for: Dietary Adjustments to Altitude Training in Elite Endurance Athletes; Impact of a Randomized Clinical Trial With Antioxidant-Rich Foods
Source: Front Sports Act Living. 2020 Aug 26;2:106. doi: 10.3389/fspor.2020.00106 (PMC7739752; doi:10.3389/fspor.2020.00106)
Supplement: Supplementary file 1 [file Data_Sheet_1.PDF]

**Supplemental Table 1.** Energy-adjusted dietary intake in the whole population before (Sea level) and during (Altitude) the 3-week altitude training camp (2320 m).

| | **All** (n=31) | | |
|---|---|---|---|
| | Sea level | Altitude | $p_{paired}$ |
| Carbohydrate (g/MJ) | 30.7 ± 2.8 | 36.4 ± 22.1 | 0.16 |
| Starch (g/MJ) | 14.1 ± 2.9 | 13.9 ± 7.2 | 0.83 |
| Mono- and disaccharides (g/MJ) | 12.0 ± 2.7 | 15.4 ± 11.7 | 0.13 |
| Added sugar (g/MJ) | 4.1 (9.6) | 3.8 (27.7) | 0.33 |
| Fiber (g/MJ) | 2.6 ± 0.6 | 2.8 ± 1.6 | 0.53 |
| Protein (g/MJ) | 10.5 ± 1.1 | 11.8 ± 6.3 | 0.16 |
| Fat (g/MJ) | 8.6 (4.2) | 7.7 (23.2) | 0.014*$^{W}$ |
| SFA (g/MJ) | 3.2 (2.8) | 3.0 (8.5) | 0.74 $^{W}$ |
| MUFA (g/MJ) | 3.0 ± 0.6 | 2.6 ± 1.0 | 0.09 |
| PUFA (g/MJ) | 1.3 ± 0.3 | 1.5 ± 1.1 | 0.55 |
| Trans fat (g/MJ) | 0.1 (12.1) | 0.1 (2.8) | 0.60 $^{W}$ |
| ω3 PUFA (g/MJ) | 0.3 (0.7) | 0.2 (1.2) | 0.023*$^{W}$ |
| ω6 PUFA (g/MJ) | 1.0 ± 0.2 | 1.6 ± 1.1 | 0.33 |
| Alcohol (g/MJ) | 0.04 (2.05) | 0.02 (0.55) | 0.003*$^{W}$ |
| Vitamin A (RAE/MJ) | 78 (252) | 49 (95) | <0.001*$^{W}$ |
| Retinol (µg/MJ) | 51 (201) | 33 (74) | <0.001*$^{W}$ |
| β-carotene (µg/MJ) | 214 (789) | 196 (625) | <0.001*$^{W}$ |
| Vitamin D (µg/MJ) | 0.8 (3.4) | 0.4 (2.4) | <0.001*$^{W}$ |
| Vitamin E (α-TE/MJ) | 1.5 ± 0.5 | 1.3 ± 0.6 | 0.20 |
| Thiamin (mg/MJ) | 0.21 ± 0.04 | 0.29 ± 0.17 | 0.017* |
| Riboflavin (mg/MJ) | 0.20 ± 0.09 | 0.18 ± 0.07 | 0.08 |
| Niacin (mg/MJ) | 2.27 ± 0.54 | 2.74 ± 1.39 | 0.07 |
| B6 (mg/MJ) | 0.21 ± 0.05 | 0.27 ± 0.16 | 0.05 |
| B12 (µg/MJ) | 0.66 ± 0.20 | 0.47 ± 0.11 | <0.001* |
| Folic acid (µg/MJ) | 29.2 ± 7.7 | 32.3 ± 20.7 | 0.43 |
| Vitamin C (mg/MJ) | 13.1 ± 7.6 | 18.1 ± 16.6 | 0.13 |
| Iron (mg/MJ) | 1.2 (5.2) | 1.1 (4.5) | 0.62 $^{W}$ |
| Calsium (mg/MJ) | 106 ± 22 | 83 ± 26 | 0.001* |
| Sodium (mg/MJ) | 248 ± 76 | 331 ± 114 | 0.001* |
| Potassium (mg/MJ) | 414 ± 75 | 460 ± 252 | 0.34 |
| Magnesium (mg/MJ) | 42.2 ± 7.2 | 46.4 ± 24.5 | 0.37 |
| Zinc (mg/MJ) | 1.3 ± 0.2 | 1.4 ± 0.6 | 0.37 |
| Selenium (µg/MJ) | 6.3 ± 2.2 | 5.6 ± 2.3 | 0.27 |
| Iodine (µg/MJ) | 14.7 ± 5.2 | 9.8 ± 3.2 | <0.001* |
| Copper (mg/MJ) | 0.1 ± 0.0 | 0.2 ± 0.1 | 0.24 |
| Phohshorus (mg/MJ) | 197 ± 29 | 186 ± 76 | 0.48 |

*Values are presented as mean ± std or median (range) for non-normally distributed data. * indicates significant difference, $p<0.05$. The p-value, $p_{paired}$ is obtained from paired tests testing the change from pre-altitude to altitude for the total population (All) either by paired t-test or Wilcoxon ($^{W}$) test. Abbreviations: SFA=saturated fat, MUFA=monounsaturated fat, PUFA=polyunsaturated fat.*
