# Supplementary material for: Dietary Adjustments to Altitude Training in Elite Endurance Athletes; Impact of a Randomized Clinical Trial With Antioxidant-Rich Foods
Source: Front Sports Act Living. 2020 Aug 26;2:106. doi: 10.3389/fspor.2020.00106 (PMC7739752; doi:10.3389/fspor.2020.00106)
Supplement: Supplementary file 2 [file Data_Sheet_2.PDF]

Supplemental Table 2. Dietary intake before (Pre-altitude) and during (Altitude), and nutrition-related blood parameters before (Pre-altitude) and after (Post-altitude) the 3-week altitude training camp (2320m), analyzed by sex (females, males).

	Females (n = 8)		Males (n = 23)		
**Dietary intake**	Pre-altitude	Altitude	Pre-altitude	Altitude	p_{change}
Energy (MJ)	12.4 ± 4.5	15.3 ± 6.4	18.5 ± 3.8	25.1 ± 3.1	0.009
Water (L)	3.7 ± 1.2	5.1 ± 0.9	5.0 ± 1.0	6.9 ± 1.1	0.194
Number of meals	5.9 ± 1.0		5.9 ± 1.0	6.5 ± 0.8	0.623
Macronutrients		6.4 ± 0.7			
Carbohydrate (g)	374 ± 120	564 ± 157	569 ± 127	813 ± 134	0.192
Carbohydrate (g/kg BW)	5.7 ± 1.8	8.6 ± 2.4	6.8 ± 1.8	9.6 ± 2.0	0.856
Carbohydrate intake during exercise > 90 min (g/h)	1 (24)	15 (26)	16 (53)	25 (44)	0.842 ^{MW}
Starch (g)	168 ± 47	207 ± 65	260 ± 71	319 ± 58	0.320
Mono- and disaccharides (g)	151 ± 71	252 ± 88	224 ± 78	329 ± 79	0.881
Added sugar (g)	48 (126)	65 (112)	70 (222)	97 (209)	0.520 ^{MW}
Fiber (g)	34 ± 7	48 ±11	46 ± 12	59 ± 13	0.953
Protein (g)	128 ± 45	181 ± 34	193 ± 37	268 ± 32	0.148
Protein (g/kg BW)	2.0 ± 0.7	2.8 ± 0.5	2.3 ± 0.5	3.2 ± 0.6	0.519
Fat (g)	112 ± 52	126 ± 42	158 ± 42	196 ± 29	0.086
SFA (g)	41 ± 22	48 ± 16	58 ± 19	78 ± 15	0.074
MUFA (g)	40 ± 20	38 ± 14	55 ± 18	62 ± 12	0.123
PUFA (g)	18 ± 6	23 ± 11	24 ± 8	32 ± 9	0.549
Trans fat (g)	1.1 (1.6)	1.0 (1.2)	1.3 (2.3)	1.8 (77)	0.132 ^{MW}
Omega-3 (g)	5.0 ± 2.9	4.2 ± 2.7	5.2 ± 3.1	5.5 ± 2.3	0.448
Omega-6 (g)	12.8 ± 4.4	18.0 ±7.9	18.9 ± 6.2	25.8 ±7.3	0.642
Cholesterol (g)	453 ± 214	469 ± 127	564 ± 235	688 ±187	0.321
Alcohol (g)	0.6 (7)	0.0 (9)	0.7 (30)	0.6 (15)	0.611 ^{MW}
Micronutrients					
Vitamin A (RAE)	1347 ± 780	949 ± 352	1675 ± 1011	1301 ± 378	0.952
Retinol (µg)	874 ± 649	594 ± 218	1254 ± 952	886 ± 360	0.793
β-carotene (mg)	3.8 (11.1)	3.3 (6.3)	3.3 (8.4)	4.9 (6.8)	0.275 ^{MW}
Vitamin D (µg)	19.7 ± 13.0	12.9 ± 13.9	18.5 ± 16.1	12.9 ± 10.2	0.798
Vitamin E (α-TE)	19.0 ± 8.4	23.5 ± 8.7	26.9 ± 10.5	28.8 ± 6.6	0.424
Thiamin (mg)	2.6 ± 0.9	4.5 ± 1.0	3.8 ± 1.1	6.3 ± 0.9	0.191
Riboflavin (mg)	2.5 ± 0.9	3.0 ± 0.8	3.7 ± 1.0	3.9 ± 0.7	0.364
Niacin (mg)	29.5 ± 11.5	41.5 ±7.6	40.3 ± 1.6	61.8 ± 6.3	0.033
B6 (mg)	2.8 ± 1.2	4.3 ± 0.8	3.7 ± 1.0	5.9 ± 1.0	0.142
B12 (µg)	8.6 ± 3.9	7.8 ± 3.1	12.0 ± 4.6	11.2 ± 2.4	0.955
Folic acid (µg)	384 ± 130	552 ± 148	511 ± 141	675 ± 123	0.060
Vitamin C (mg)	154 (243)	345 (268)	184 (671)	335 (339)	0.740 ^{MW}
Iron (mg)	16.0 (15.0)	18.4 (12.0)	21.1 (70)	28.2 (130)	0.048 ^{MW}
Calsium (mg)	1323 (569)	1703 (1955)	1897 (2258)	1870 (1682)	0.317 ^{MW}
Sodium (mg)	3040 ± 1988	4509 ± 1725	4535 ± 1385	8174 ± 2047	0.015
Potassium (mg)	5230 ± 1779	7602 ±1646	7406 ± 1605	9970 ± 1605	0.771

Magnesium (mg)	521 ± 187	721 ± 173	772 ± 207	1037 ± 193	0.437
Zinc (mg)	15.4 ± 5.3	21.1± 4.2	23.4 ± 5.4	031.0 ± 3.4	0.377
Selenium (µg)	80.3 ± 29.6	88.4 ±19.9	113.4 ± 51.4	127.5 ± 23.5	0.761
Iodine (µg)	183 (300)	152 (228)	258 (335)	210 (321)	0.808 [MW]
Cobber (mg)	1.8 ± 0.6	2.6 ± 0.7	2.5 ± 0.6	3.5 ± 0.5	0.556
Phohsphorus (mg)	2426 ± 764	3082 ± 645	3602 ± 923	4161 ± 572	0.766
**Blood parameters**	**Pre-altitude**	**Post-altitude**	**Pre-altitude**	**Post-altitude**	**p_{change}**
s-vitamin D	94 ± 23	85 ± 18	115 ± 20	96 ± 12	0.294
s-vitamin E	24.1 ± 5.5	27.9 ± 8.5	24.9 ± 5.8	27.0 ± 5.8	0.597
s-folate	23.1 ± 7.0	25.1 ± 9.3	20.6 ± 7.0	19.2 ± 5.8	0.240
s-B12	402 ± 211	388 ± 173	336 ± 100	339 ± 88	0.745
s-iron	21.1 ± 7.7	11.7 ± 4.2	21.3 ± 7.1	18.8 ± 5.5	0.034
s-ferritin	43 ± 13	47 ± 46	107 ± 64	86 ± 57	0.049
s-ReHb	33.4 ± 1.0	33.9 ± 1.5	34.0 ± 1.2	34.6 ± 1.1	0.988
s-LDL-c	2.5 ± 0.6	2.5 ± 0.6	2.6 ± 0.7	2.7 ± 0.9	0.457
s-HDL-c	1.8 ± 0.3	1.6 ± 0.2	1.6 ± 0.4	1.5 ± 0.4	0.755
s-SHBG	56.3 ± 20.5	54.0 ± 13.9	35.7 ± 12.0	38.2 ± 15.2	0.306
s-testosterone	*na*	*na*	14.3 ± 5.6	17.1 ± 6.2	0.052[1]
s-oestradiol	0.25 ± 0.09	0.28 ±0.15	*na*	*na*	0.523[2]

Values are presented as mean ± std or median (range) for non-normally distributed data. The p-value, pchange is obtained from comparing the change (from Pre-altitude to Altitude) between the males and females using t-test or Wilcoxon-MW tests depending on the normality of the data. 1The p-value is obtained from paired tests testing the change from Pre-altitude to Altitude in males by paired t-test. 2The p-value is obtained from paired tests testing the change from Pre-altitude to Altitude in females by paired t-test.Abbreviations: BW=body weight, HDL-c=high density lipoprotein cholesterol, LDL-c = low density lipoprotein cholesterol, MUFA=monounsaturated fat, PUFA=polyunsaturated fat, ReHb=Reticulocyte hemoglobin, SFA=saturated fat, SHBG = sex hormone binding globulin.
